# Impact of feeding habits on the development of language-specific processing of phonemes in brain: An event-related potentials study

**DOI:** 10.3389/fnut.2023.1032413

**Published:** 2023-02-17

**Authors:** Graciela C. Alatorre-Cruz, Aline Andres, Yuyuan Gu, Heather Downs, Darcy Hagood, Seth T. Sorensen, David Keith Williams, Linda J. Larson-Prior

**Affiliations:** ^1^Department of Pediatrics, University of Arkansas for Medical Sciences, Little Rock, AR, United States; ^2^Arkansas Children’s Nutrition Center, Little Rock, AR, United States; ^3^Department of Biostatistics, University of Arkansas for Medical Sciences, Little Rock, AR, United States; ^4^Departments of Neurobiology and Developmental Sciences, Psychiatry, Neurology, Pediatrics and Biomedical Informatics, University of Arkansas for Medical Sciences, Little Rock, AR, United States

**Keywords:** infancy, infant’s diet, language development, stimuli awareness, MMN

## Abstract

**Introduction:**

Infancy is a stage characterized by multiple brain and cognitive changes. In a short time, infants must consolidate a new brain network and develop two important properties for speech comprehension: phonemic normalization and categorical perception. Recent studies have described diet as an essential factor in normal language development, reporting that breastfed infants show an earlier brain maturity and thus a faster cognitive development. Few studies have described a long-term effect of diet on phonological perception.

**Methods:**

To explore that effect, we compared the event-related potentials (ERPs) collected during an oddball paradigm (frequent /pa/80%, deviant/ba/20%) of infants fed with breast milk (BF), cow-milk-based formula (MF), and soy-based formula (SF), which were assessed at 3, 6, 9, 12, and 24 months of age [Mean across all age groups: 127 BF infants, Mean (*M*) 39.6 gestation weeks; 121 MF infants, *M* = 39.16 gestation weeks; 116 SF infants, *M* = 39.16 gestation weeks].

**Results:**

Behavioral differences between dietary groups in acoustic comprehension were observed at 24-months of age. The BF group displayed greater scores than the MF and SF groups. In phonological discrimination task, the ERPs analyses showed that SF group had an electrophysiological pattern associated with difficulties in phonological-stimulus awareness [mismatch negativity (MMN)-2 latency in frontal left regions of interest (ROI) and longer MMN-2 latency in temporal right ROI] and less brain maturity than BF and MF groups. The SF group displayed more right-lateralized brain recruitment in phonological processing at 12-months old.

**Discussion:**

We conclude that using soy-based formula in a prolonged and frequent manner might trigger a language development different from that observed in the BF or MF groups. The soy-based formula’s composition might affect frontal left-brain area development, which is a nodal brain region in phonological-stimuli awareness.

## 1. Introduction

In infant development, the brain undergoes multiple changes, including increased myelination and configuration of synaptic connections needed to consolidate new brain networks. Volumetric brain growth, which proceeds through infancy, reaches adult levels at 3 years old. These changes are promoted by environmental stimuli (1), hormonal status and genetic factors (2). Moreover, infant diet has been recently recognized as an important contributor to cognitive development, immune system development, and healthy physical growth (3–6). To support infant development, the diet should provide micro and macronutrients such as docosahexaenoic (DHA) and arachidonic (AA), long-chain fatty acids, lutein, choline, and hormones (7–9). Human milk provides these essential nutrients (5, 6) and promotes greater brain maturity characterized by healthier neuronal growth and myelination, and greater infant gray and white matter (8, 10–12).

Some studies report that breastfed infants show an earlier development of language perception (10, 13–15) and memory than those fed with nutrient-enriched formula (16). An explanation for this finding is that human milk changes in composition from colostrum to late lactation, and varies by the mother’s biological condition, while milk-based formula maintains a stable composition (5, 9, 10). In particular, human milk seems to have a better nutritional composition than milk-based formula because: (1) complex oligosaccharides or lipid components such as gangliosides found in human milk are not available in milk-based formula composition or have not been clinically proven (17), and (2) the equilibrium in human milk’s composition between DHA, lutein, choline (10, 16), and complex oligosaccharides seems to promote better cognition (18, 19). Therefore, differences in the proportions of formula components may negatively affect infant’s nutrition, and consequently, the infant’s cognitive development (20).

In the first year of life, phonological perception should be developed; otherwise, the infant will suffer delayed language development (21). This milestone entails the fast growth of multiple brain areas regulated by healthy nutritional habits, particularly microbiome is essential in synaptogenesis and metabolic brain requirements, affecting infant brain development and behavior (22, 23). Moreover, recent studies suggest that breastfeeding positively affects cognition and brain development compared with other feeding habits (24–28). They explain that this effect occurs because four reasons: (1) human milk might make a difference in brain structure and function *via* fatty acids, affecting cell membranes and influencing gene expression within these cells, (2) human milk contains a variety of constituents that promote optimal development, (3) the relationship between the immune system and breastfeeding might influence learning and memory, and (4) lactation affects mothers’ way they teach the language (29).

Even *in utero*, infants are able to distinguish between sounds (30–32) and show habituation to repetitive stimuli (30). However, they must develop other abilities to reach adult levels of phonological perception. Within the first 2 months of life, normal infants show a precognitive detection of syllable length (33); at 4 months they begin to distinguish between tones and syllables (34). At 6 months old, they establish prototypes of vowels in their native language (31, 32), and around 10 months old, infants have prototypes of consonants (35). Between 9 and 10 months of age, infants can distinguish words (36), and preserve the detection of foreign-language contrast until 11 months old (37). By the end of the first year of life, they have access to phonological representations akin to those of adults, that is, the infant has developed two important properties for speech comprehension: (1) phonemic normalization and (2) categorical perception (30). These subtle behavioral changes are accompanied by the recruitment of frontal and temporal lobes responsible for phonological perception and semantic categorization, which are differentially developed in the first year of life (38, 39). In infants, brain maturity is reflected in the decrease of bilateral brain responses and increase in left lateralization of brain activity (40–42), culminating in the development of the adult pattern of dorsal and ventral pathways associated with language function (43–45).

Accordingly, brain-electrical activity associated with phonological perception also develops during infancy, reflecting the increasing ability to decode incoming speech supported by the accurate perception of rapid acoustic changes (21, 46, 47). The brain-electrical response to auditory tones [event-related potentials (ERPs)] in adults comprises the P1-N1-P2-N2 complex (48–50), and includes (1) a positive deflection at 150 ms on fronto-central sites (P150 or P1), which has been associated with features of acoustic stimulus (51–53) and modulated by an inter-stimulus interval (54); (2) a negative deflection at 250 ms (N250 or N1) and another at 450 ms (N450 or N2), which reflect the differences between acoustic stimulus (e.g., such as complexity and frequency) (54), and (3) a positive wave at 350 ms (P350 or P2) which has been associated with stimulus awareness and perceptual salience, and is commonly identified as an index of auditory recognition memory (51). However, these ERP components are not exhibited at birth, but develop gradually over the first year of life. At birth, infants display a large positive wave between 100 and 450 ms followed by the N2 component (49). At 3 months old, the positive wave is divided by the N1 component between 160 and 200 ms (49, 55), resulting in two ERP components: a P1 and P2, and the amplitude of these components seem to increase over the next month (49, 54). Between 3 and 6 months the amplitude of N1 and N2 components increase (56), and exhibit the P1-N1-P2-N2 complex. This ERP morphology is maintained until 2 years of age (49, 57), with an increase in component amplitude exhibited at 12-months old (49). Although few studies describe the functional significance of these ERP components in infancy, it has been speculated they have similar function to those observed in children and adults (53).

Development of phonemic perception requires infants to detect differences in acoustic features and phonological categories, leading to the use of experimental auditory oddball paradigms in which stimuli including differences between acoustic features, frequency or phonological categories are especially useful in assessing brain electrical activity associated with the acquisition of language (32–34, 58–61). From studies in children and adults, the expectation is that the amplitude of P1-N1-P2-N2 complex will be greater for uncommon than common repetitive stimuli (50), due to the fact that neuronal responses habituate to repeated presentation of the same stimulus, while a new, unusual stimulus will produce a large amplitude response (30, 62). The difference between the conditions is called mismatch negativity (MMN) (30, 63, 64). It has been reported that two MMNs which appear at 6 months (50, 54), correspond to the differences in P1 and P2 components (65). As described above, the MMN components undergo latency decreases with increasing age (50). In addition, the MMN components have been linked to the computation of acoustic features such as duration or intensity (66, 67), arbitrary rules (68), or lexical and grammatical status (58, 69), and their interpretation depends on the specific stimulus type presented.

While few studies have assessed how diet affects phonetic perception; those that did have shown variations on this cognitive process by diet. Li et al. (13) compared breastfed infants and infants fed with soy or cow-milk-based formula in their phonological perception at 3 and 6 months, using an oddball paradigm compromised of frequent and deviant syllables (/pa/standard and/ba/deviant). The authors found an advanced neural maturation in breastfed infants characterized by a greater P350/P2 amplitude in frontal regions at 3 months, and shorter P2 latency at 6 than 3 months old than the other dietary groups. Using the same paradigm, Pivik et al. (3) compared these same dietary groups and ages. However, they did not replicate the findings of Li et al. (13), reporting no age-related changes in ERP components. In this study, differences were related only to diet group, with breastfed infants displaying shorter P1 latencies and smaller P1 amplitude for deviant rather than standard stimuli than infants fed with soy milk. The authors interpreted that to indicate that breast-fed infant show more rapid encoding of acoustic information than the other diet groups. The same diet groups were also studied at 4 and 5 months (14), where changes in P350/P2 amplitude across age for each syllable, depended on the diet. Infants fed with soy milk showed a decreased P2 amplitude for deviant stimuli than the other groups, while the breastfed infants displayed decreased amplitude for standard stimuli compared with other dietary groups. The authors concluded that diet affects attention and memory functions involved in the processing and discrimination of speech sounds.

The primary aim of the present study was to determine the differences in phonological perception assessed by electrophysiological response to frequent and deviant phonemes at 3, 6, 9, 12, and 24 months between three dietary groups: breast fed (BF), cow-milk-formula fed (MF), and soy-formula fed (SF) infants. As previous studies have reported evidence for earlier phonological perception in BF infants (13, 14, 65), we anticipated that the BF group would show (1) greater amplitude and shorter latency of MMN components than MF and SF groups, (2) greater amplitude and shorter latency of MMN components (13, 65) at 6 month-old when the P1-N1-P2-N2 complex reaches a stable morphology (49, 57), and at 12 months when ERP amplitudes have a stable morphology (49) and (3) greater hemispheric asymmetry of MMN components (40).

## 2. Materials and methods

### 2.1. Participants

The study included full-term infants between 3 and 24 months old. All of them had a birth weight of over 3 kg and were a product of uncomplicated pregnancies; the mothers reported no medical diagnoses during pregnancy or lactation. Mothers with alcohol, tobacco, or medication use were excluded. In this longitudinal study, 2-month-old infants were stabilized on one of three diets which were selected by parents: BF, MF, and SF, the two last fortified with DHA and AA. Each infant was provided the same diet until 12 months of age. The infants were assessed at 3, 6, 9, 12, and 24 months old, resulting in 15 groups of data (e.g., subjects aged at three-months-old distributed into three groups: BF, MF, and SF). Socioeconomic status [SES, measured by the Four-Factor Index of Social Positions (70)] of the infants’ parents was collected at the beginning of this study. The infants’ anthropometric measures (i.e., height, weight, and head circumference) and food intake history were collected at each visit. Infants and mothers underwent neuropsychological and psychophysiological testing, which was conducted by a certified examiner. The mother’s assessment included Wechsler the Abbreviated Scale of Intelligence [WASI-II, (71)] and Symptoms Assessment-45 questionnaire [SA-45, (72)], while infants were evaluated using the Bayley Scales of Infant and Toddler Development [BSID-2, (73)], Preschool Language Scale [PLS-3, (74)] as well as the psychophysiological oddball paradigm to assess phonological-discrimination. Most of the parents reported English as their language at home (see Table 1). All mothers reached an Intelligence quotient (IQ) score higher than 70 on the WASI-II test. Participants were excluded from this study if they did not complete all assessments. The protocol was approved by the Institutional Review Board of the University of Arkansas for Medical Sciences. Informed consent was obtained from parents.

**Table 1:** Characteristics of dietary groups.

Age	Total (*n*)	Type of diet (*n*)	Mother IQ mean (SD)	Language in home
3 m	410	BF: 137	BF: 109.4 (10.2)	E (404)
		MF:138	MF: 105.8 (9.2)	S (1)
		SF: 135	SF: 102.9 (11.7)	E,S (4)
				E,O (1)
6 m	365	BF: 119	BF: 110.1 (10.1)	E (356)
		MF:126	MF: 105.7 (9.3)	E,S (7)
		SF: 120	SF: 103.3 (10.0)	E,O (2)
9 m	340	BF: 113	BF: 109.6 (10.5)	E (333)
		MF:114	MF: 105.4 (8.7)	S (1)
		SF: 113	SF: 103.8 (10.3)	E,S (5)
				E,O (1)
12 m	334	BF: 122	BF: 109.7 (10.3)	E (318)
		MF:112	MF: 105.0 (8.8)	S (2)
		SF: 100	SF: 103.2 (10.3)	E,S (10)
				E,O (4)
24 m	372	BF: 142	BF: 109.6 (10.4)	E (361)
		MF:117	MF: 105.8 (9.1)	S (1)
		SF: 113	SF: 104.5 (10.7)	E,S (7)
				E,O (3)

m, months; n, number of participants; BF, breast fed; MF, milk fed; SF, soy fed; IQ, intelligence quotient from Wechsler Abbreviated Scale of Intelligence (WASI-II); E, English; S, Spanish; O, other languages (i.e., Swedish, Arabic, and Chinese).

### 2.2. Experimental design

**Figure 1:**
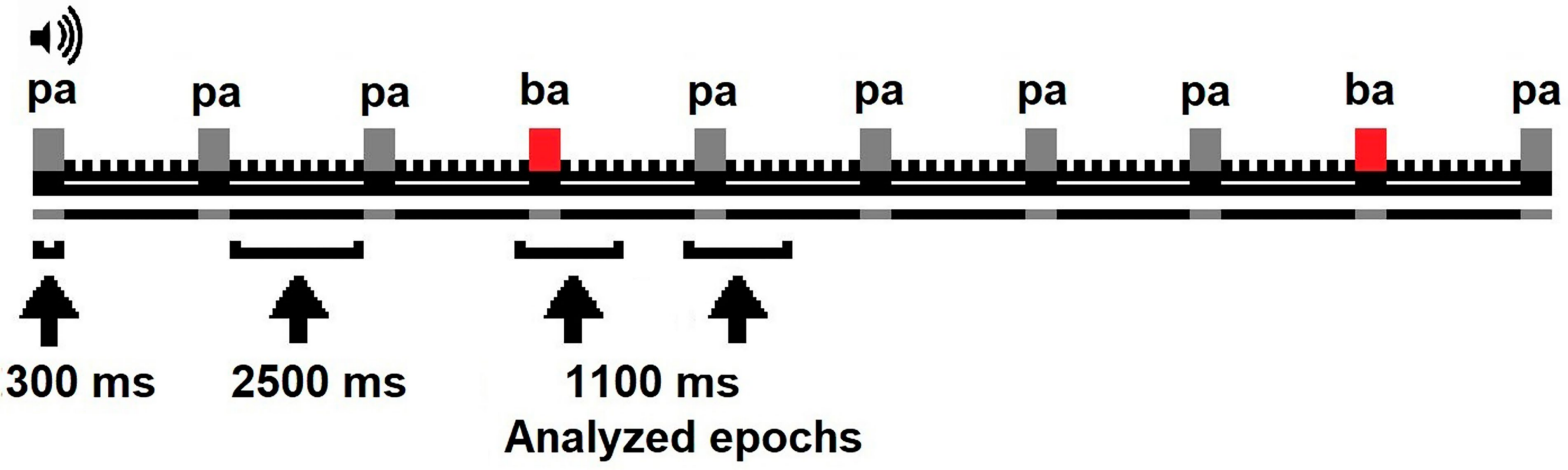
Oddball paradigm applied to infants.

Phonological discrimination was assessed using an auditory-oddball paradigm while an electroencephalogram (EEG) was recorded. The infants were awake and seated in their parent’s lap or infant chair in a sound-isolated, shielded recording chamber. Silent videos were played to engage the infant’s attention. The paradigm was constituted of two types of stimuli, one of them was frequent (/pa/80%) and the other deviant (/ba/20%). Both stimuli were syllables with consonant-vowel structure, had the same intensity (72 dB SPL), and were pronounced by a native English speaker through speakers located at 5 ft. from the infant. The stimuli were designed and administered using E-Prime software (version 1). All stimuli appeared during 300 ms with a stimulus onset asynchrony (SOA) of 2,500 ms. The SOA was selected because longer intervals attenuate standard-deviant response differences (75) and exceeds the limits of sensory memory reported for infants (76). The task included three blocks of 90 trials for a total of 270 trials. The deviant stimuli (/ba/) randomly appeared in each block with a probability of 0.2. Each block lasted 4.2 min. The infants had two rest periods of 5 min between experimental blocks (see Figure 1).

### 2.3. Data acquisition/prep-processing

The EEG was acquired with a Geodesic Net Amps 200 system running Netstation 2 software using the 128-channel (Electrical Geodesics, Inc., Eugene OR, United States). Data were amplified with a bandpass of 0.1–100 Hz and a sampling rate of 250 Hz. Electrode impedances were kept below 40 kΩ. Eye movements and blinks were monitored. Data were analyzed offline using the Matlab toolbox (Matlab version R2020a). The EEG was segmented into epochs with a 100 ms pre-stimulus baseline and 1,000 ms stimulus/post-stimulus. The epochs were subjected to an automatic artifact detection algorithm. Bad channels (i.e., channels with fast average amplitude greater than 200 μV or/and differential average greater than 100 μV) were interpolated from nearby good channels using spherical splines. Trials with more than 10 bad channels were excluded. The accepted segments for each type of condition (/ba/or/pa/) were baseline corrected using a 100 ms pre-stimulus time window, re-referenced to the common mean, and averaged for each participant. The accepted segments were at least 35 per condition for each participant.

### 2.4. Event-related potentials

**Figure 2:**
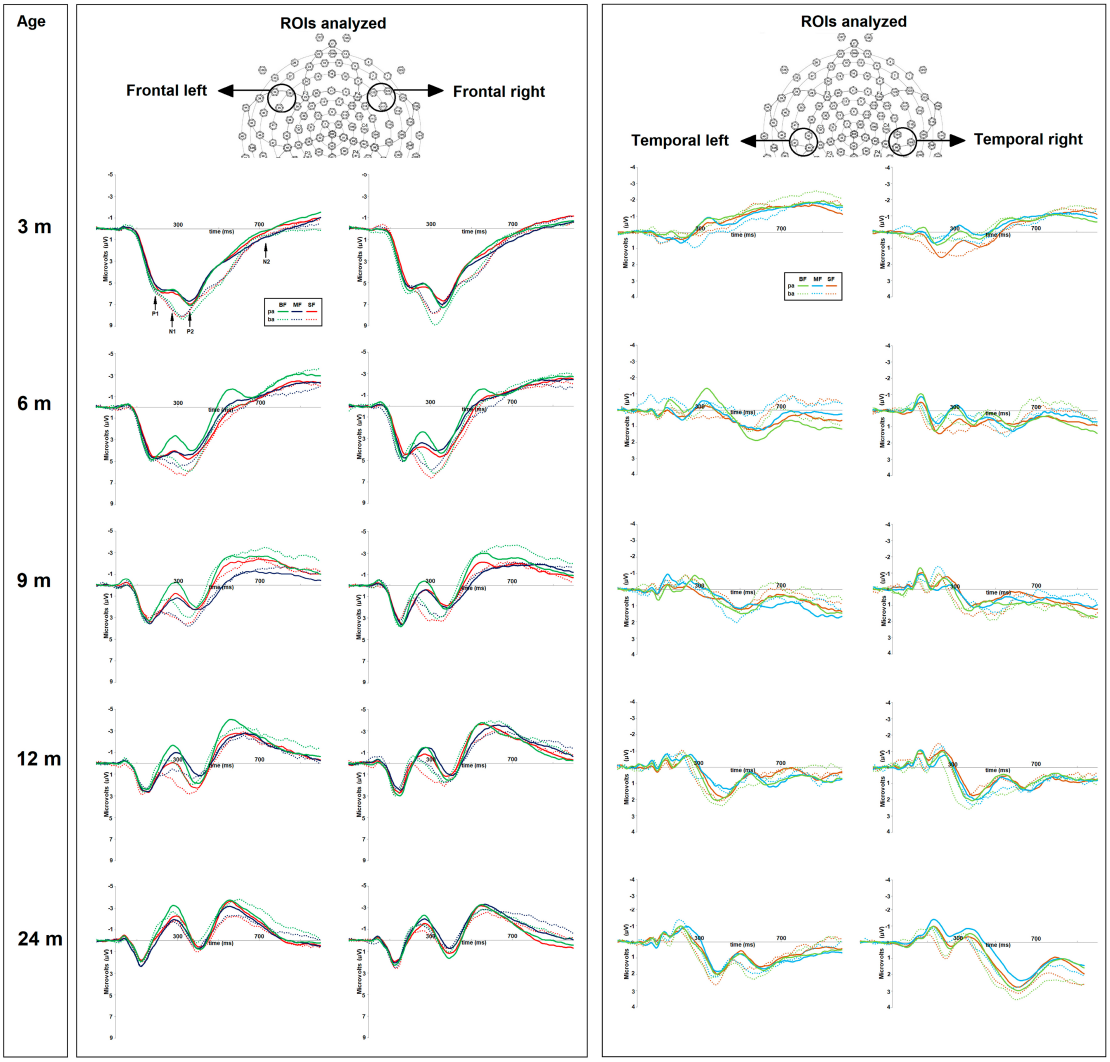
On the top, the regions of interest (ROIs) used for amplitude and latency analyses of ERP components. On the bottom, the grand average of ERPs of frequent “pa” and deviant “ba” conditions for each dietary group (BF, breast feed; MF, milk feed; SF, soy feed) at 3, 6, 9, 12, and 24-months old. The positive or negative event-related potentials (ERP) components were highlighted as follows: P1-N1, P2, and N2.

The average epoch for each condition per subject was obtained in four regions of interest (ROIs): Frontal Left (FL; sensors 28, 34, and 35) and Right (FR; sensors 117, 122, and 123), Temporal Left (TL; sensors 42, 47, and 48) and Right (TR; sensors 99, 103, and 104) (see Figure 2). Then, the difference wave was calculated in each ROI by subtracting the epoch associated with the frequent stimulus (/pa/) from that related to the deviant stimulus (/ba/). The grand average of difference wave was inspected in accordance with the ERP literature associated with phonological perception (30, 54, 63, 64). Two ERPs components were identified, two mismatch negativities; the first between 75 and 255 ms (MMN-1), and the second between 300 and 500 ms (MMN-2), the first functionally associated with the P1 component and the second with the P2 component.

### 2.5. Data analysis methods

#### 2.5.1. Characteristics of dietary groups and behavioral data

##### 2.5.1.1. Parental data

Parental SES in each age group (i.e., 3, 6, 9, 12, and 24-months old) was compared using one-way ANOVA. For both comparisons, dietary group (i.e., BF, MF, and SF) was included as a between-subjects factor, and total SES index was included as within-subject factors.

Maternal psychometric and psychiatric data: Psychometric and psychiatric test results were analyzed using two-way ANOVAs for each assessment (i.e., WASI-II and SA-45) and each age group. The dietary group was included as a between-subjects factor, and the within-subject factors were as follows:

- WASI-II is a test that estimates the general intellectual ability by measuring verbal, nonverbal, and general cognition of adults; this test consists of two indices: Perceptual reasoning index (PRI) and Verbal comprehension index (VCI). The indices were included as within-subjects factors.
- SA-45 is a questionnaire that is constituted by two indices designed to assess general psychiatric symptomatology. The indices are the Global severity index (GSI) and Positive symptom Index (PST); these were included as within-subjects factors.

##### 2.5.1.2. Infants data

###### 2.5.1.2.1. Infants’ anthropometric and psychometric data

Birth data and anthropometric measures were compared at 3, 6, 9, 12, and 24-months old using one-way ANOVA. For both comparisons, the dietary group was included as a between-subjects factor, and gestational age, birth length, birth weight, height, weight, and head circumference were separately included as between-subjects factors. A chi-squared test was used to compare groups for infant’s sex distribution.

Psychometric test results were analyzed using two-way ANOVAs for each neuropsychological assessment (i.e., BSID-2 and PLS-3 tests) and for each age group. The dietary group was included as a between-subjects factor, and within-subject factors are as follows:

- BSID-2 is a standard series of measurements used to assess the infant’s development between one and 42 months, and it is constituted by Mental development index (MDI) and psychomotor development index (PDI). Both were included as within-subject factors.
- PLS-3 is a test used to assess receptive and expressive language skills in infants. This consists of two subscales: auditory comprehension (AC) and expressive communication (EC); these subscales were considered as a within-subject factor.

###### 2.5.1.2.2. Infant’ amplitude and latency analyses of ERPs

Comparisons between dietary groups for each age group: We considered MMN-1 and MMN-2 components for the statistical analyses. We calculated the mean amplitude and its latency (i.e., the maximal peak of time window) for each ERP component. Then, we separately compared the amplitude and latency of each ERP component. ANCOVAs were also separately computed for each age group. The dietary group was the between-subject factor, FL, FR, TL, and TR ROIs were included as the within-subject factors, and gestational weeks and infant’s sex as covariables.

We also assessed the hemispheric asymmetry of ERPs components, ANCOVAs were separately computed for the difference in amplitude or latency of ERPs between brain hemispheres in frontal or temporal regions (e.g., MMN-1 amplitude in frontal left ROI minus MMN-1 amplitude in frontal right ROI). The dietary group was the between-subject factor, frontal and temporal ROIs were included as the within-subject factors, and gestational weeks and infant’s sex as covariables.

Comparisons between age groups for each dietary group: ANCOVAs were separately performed for the amplitude or latency of each ERP component and each dietary group. The age group (3, 6, 9, 12, and 24 months) was the between-subject factor, FL, FR, TL, and TR ROIs were included as the within-subject, and gestation weeks and infant’s sex as covariables. Data were analyzed using SPSS Statistics 20 and Matlab (version R2020a). Greenhouse–Geisser corrections were made for violations of sphericity when the numerator was greater than 1. value of *p*s resulting from a set of comparisons were corrected by the FDR method. We report results surviving FDR correction (*p*-values <0.05).

##### 2.5.1.3. Regression analyses

Regression analyses were performed to identify the association between amplitude and latency of ERP components in each ROI, that differed between dietary groups, and those factors that might explain the variability in the brain-electrical activity. The linear regression included amplitude or latency in FL, FR, TL or TR ROIs as the dependent variables, with dietary group (i.e., BF, MF, and SF), mom’s cognitive and psychiatric status (WASI-II: PRI and VCI; SA-45: GSI and PST), gestation weeks, infant’s sex, PLS-3: AC and EC subscales as the independent variables. Linear regressions were performed by age group. The linear regression analyses included multiple-linear backward regressions to find a reduced model that best explains the data.

Regression analyses were also performed to identify the association between the hemispheric asymmetry of ERPs components and other variables. Hemispheric differences in frontal or temporal regions were included as dependent variables, and the independent variables were dietary group (i.e., BF, MF, and SF), mom’s cognitive and psychiatric status (WASI-II: PRI and VCI; SA-45: GSI and PST), gestation weeks, infant’s sex, PLS-3: AC and EC subscales. Linear regressions were performed by age group. The linear regression analyses included multiple-linear backward regressions to find a reduced model that best explains the data. Factors with the highest *value of p* were removed until all factors were statistically significant. A *value of p* < 0.05 was considered statistically significant in all analyses.

## 3. Results

### 3.1. Parental data

We observed a significant main effect of the dietary group at 3-months [*F*(2,398) 3.5, *p* = 0.03], and 6-months of age [*F*(2,361) 5.4, *p* = 0.005]. The *post hoc* tests showed that the BF group displayed a greater parental SES score than SF group at 3-months old [Mean difference (MD) = −2.7, *p* = 0.03; BF, Mean (*M*) 39.8; MF, *M* = 38.4; SF, *M* = 37.0], while at 6-months old, SF group was significantly different than BF and MF groups, displaying a lower parental SES score than the other groups (SF vs. BF, MD = −3.2, *p* = 0.008; SF vs. MF, MD = −2.8, *p* = 0.02; BF, *M* = 40.0; MF, *M* = 39.6; SF, *M* = 36.8).

**Figure 3:**
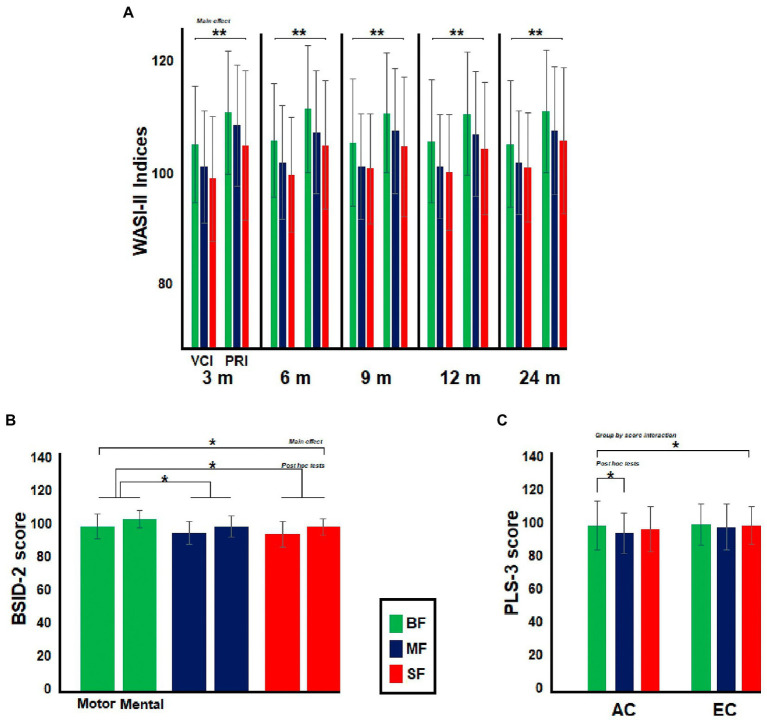
Differences between dietary groups in neuropsychological and psychophysiological assessment. In **(A)**, the bar graph illustrates differences between Infant’s moms in Wechsler Abbreviated Scale of Intelligence (WASI-II). The moms from BF group showed greater WASI-II indices than the remaining dietary groups. In **(B)**, the bar graph shows differences between the dietary groups in Bayley Scales of Infant and Toddler Development (BSID-2) at 9-months old, BF groups displayed greater BSID-2 scores than MF and SF groups, while in **(C)**, the bar graph illustrates differences between dietary groups in Preschool Language Scale (PLS-3) at 24-months old. BF infants showed greater AC score than MF group. Significant value of *p*s has been represented as follows: *p < 0.05, **p < 0.01.

The dietary groups differed in maternal WASI-II indices. In all comparisons, the *post hoc* tests showed that mothers from the BF group had greater WASI-II indices than mothers in the other dietary groups [3 months (m), BF vs. MF, MD = 3.1, *p* = 0.02; BF vs. SF, MD = 5.8, *p* < 0.001; 6 m, BF vs. MF, MD = 3.9, *p* = 0.002; BF vs. SF, MD = 6.1, *p* < 0.001; 9 m, BF vs. MF, MD = 3.7, *p* = 0.007; BF vs. SF, MD = 5.2, *p* < 0.001; 12 m, BF vs. MF, MD = 3.9, *p* = 0.003; BF vs. SF, MD = 5.7, *p* < 0.001; 24 m, BF vs. MF, MD = 3.3, *p* = 0.01; BF vs. SF, MD = 4.5, *p* < 0.001]. No significant dietary group by WASI-II indices interaction was found in any comparison (see Figure 3A).

Although no significant main effect of dietary group was observed in maternal SA-45 indices, a significant dietary group by SA-45 interaction was found at 12 months [*F*(2,326) 6.3, *p* = 0.002, Ƞ^2^ = 0.04, ε = 1]. However, the *post hoc* tests showed no significant differences between dietary groups in any SA-45 index (BF: GSI, *M* = 45.6; PST, *M* = 45.1; MF: GSI, *M* = 45.6; PST, *M* = 44.3; SF: GSI, *M* = 45.7; PST, *M* = 45.4).

### 3.2. Infant’s data

#### 3.2.1. Infant’s anthropometric and psychometric data

##### 3.2.1.1. Anthropometric data

As is shown in Table 2, gestational weeks differed between groups in all age groups, the *post hoc* tests evinced that the BF group had greater gestation weeks than the other dietary groups. The dietary groups also differed in birth weight at 6 months old. The *post hoc* test showed greater birth weight for BF than SF group (MD = 0.1, *p* = 0.02).

**Table 2:** Infant’s anthropometric measures by dietary group.

Age	Variables	Dietary group			Main effect of group	
		BF	MFMean (SD)	SF	*F*	*p*
3 m	Gestation (weeks)	39.5 (1.0)	39.1 (0.9)	39.1 (1.0)	6.9	0.001**
6 m		39.6 (1.0)	39.1 (0.9)	39.2 (1.0)	7.2	0.001**
9 m		39.6 (1.0)	39.2 (0.9)	39.2 (1.0)	6.3	0.002**
12 m		39.5 (1.0)	39.2 (0.9)	39.2 (1.1)	5.3	0.005**
24 m		39.6 (1.0)	39.2 (0.9)	39.1 (1.0)	8.8	0.000**
3 m	Birth weight (kg)	3.5 (0.3)	3.5 (0.4)	3.4 (0.4)	2.9	0.06
6 m		3.5 (0.3)	3.5 (0.4)	3.4 (0.4)	3.6	0.03*
9 m		3.5 (0.3)	3.5 (0.4)	3.5 (0.4)	1.4	0.2
12 m		3.5 (0.3)	3.5 (0.4)	3.4 (0.4)	1.8	0.2
24 m		3.5 (0.3)	3.5 (0.4)	3.4 (0.4)	2.1	0.1
3 m	Birth length (cm)	51.4 (2.2)	51.1 (2.5)	51.2 (2.2)	0.9	0.4
6 m		51.5 (2.3)	51.2 (2.4)	51.2 (2.1)	0.6	0.5
9 m		51.5 (2.1)	51.2 (2.7)	51.2 (2.0)	0.9	0.4
12 m		51.4 (2.1)	51.1 (2.5)	51.2 (2.1)	0.5	0.6
24 m		51.4 (2.2)	51.2 (2.4)	51.0 (2.10)	0.9	0.4
3 m	Height (cm)	60.1 (1.9)	58.9 (2.1)	59.7 (1.6)	1.5	0.2
6 m		66.0 (2.3)	66.4 (2.4)	66.4 (2.0)	1.5	0.2
9 m		69.9 (2.3)	70.7 (2.4)	70.8 (2.3)	5.2	0.006**
12 m		73.7 (2.3)	74.4 (2.3)	75.0 (2.3)	8.3	0.000**
24 m		86.2 (2.5)	86.3 (2.8)	86.7 (2.8)	1.3	0.3
3 m	Weight (kg)	6.1 (0.7)	6.1 (0.6)	5.9 (0.5)	2.2	0.8
6 m		7.6 (0.9)	7.9 (0.9)	7.8 (0.8)	2.8	0.06
9 m		8.6 (0.8)	9.0 (0.9)	9.1 (1.0)	10.8	0.000**
12 m		9.4 (1.0)	9.9 (0.9)	10.0 (1.0)	13.3	0.000**
24 m		12.2 (1.2)	12.4 (1.2)	12.7 (1.3)	6.3	0.002**
3 m	Head circ. (cm)	40.7 (1.1)	40.6 (1.1)	40.6 (1.1)	0.2	0.8
6 m		43.4 (1.3)	43.6 (1.1)	43.7 (1.3)	1.6	0.2
9 m		45.2 (1.2)	45.2 (1.2)	45.4 (1.3)	1.1	0.3
12 m		46.4 (1.3)	46.4 (1.2)	46.6 (1.4)	1.6	0.2
24 m		48.7 (1.3)	48.8 (1.3)	48.9 (1.4)	0.7	0.5
			F/M		*χ*^2^ (2)	*p*
3 m	Sex	68/69	71/67	62/73	0.9	0.6
6 m		64/55	55/65	62/64	1.5	0.5
9 m		58/55	55/59	49/64	1.5	0.5
12 m		66/56	56/56	43/57	2.7	0.2
24 m		74/68	56/61	57/56	0.5	0.8

m, months; BF, breast fed; MF, milk fed; SF, soy fed; SD, standard deviation; F, female; M, male; Head circ., head circumference. *p < 0.05; **p < 0.01.

The height and weight differed between dietary groups at 9 and 12 months old, with *post hoc* tests showing lower height and weight for BF infants. The comparison also revealed differences between dietary groups in weight at 24 months old, with BF infants showing lower weight than SF group (MD = −0.5, *p* = 0.001). No differences between dietary groups were found in birth length, head circumference or infant’s sex in any age group.

##### 3.2.1.2. Psychometric data

Consistent with a previous behavioral study comparing these same dietary groups (4), no differences in MDI and PDI indexes of BSID-2 test were found at 3, 12, or 24 months old. The dietary groups only differed in BSID-2 indexes at 9-months old [*F*(2,331) 3.6, *p* = 0.03, *Ƞ^2^* = 0.02] (see Figure 3B). The *post hoc* tests showed that the BF group displayed greater BSID-2 indexes than SF and MF groups (BF vs. MF, MD = 1.5, *p* = 0.04; BF vs. SF, MD = 1.8, *p* = 0.01). No significant dietary group by BSID-2 indexes interaction was found in any comparison. No significant main effect of group was observed in PLS-3 test in any age group. However, a significant dietary group by PLS-3 interaction was found at 24 months old [*F*(2,336) 3.4, *p* = 0.03, *Ƞ^2^* = 0.02, ε = 1]. The *post hoc* tests revealed that the BF group displayed a greater AC score than the MF group (MD = 4.8, *p* = 0.01; see Figure 3C).

#### 3.2.2. Infant’s ERPs analysis

Comparisons between dietary groups for each age group.

##### 3.2.2.1. Amplitude and latency of ERPs

###### 3.2.2.1.1. MMN-1 component

The dietary groups did not differ in amplitude or latency of the MMN-1 component at any age group. No significant main effect of dietary group or dietary group by ROIs interactions were observed in any comparison (see Supplementary Table S1).

###### 3.2.2.1.2. MMN-2 component

**Figure 4:**
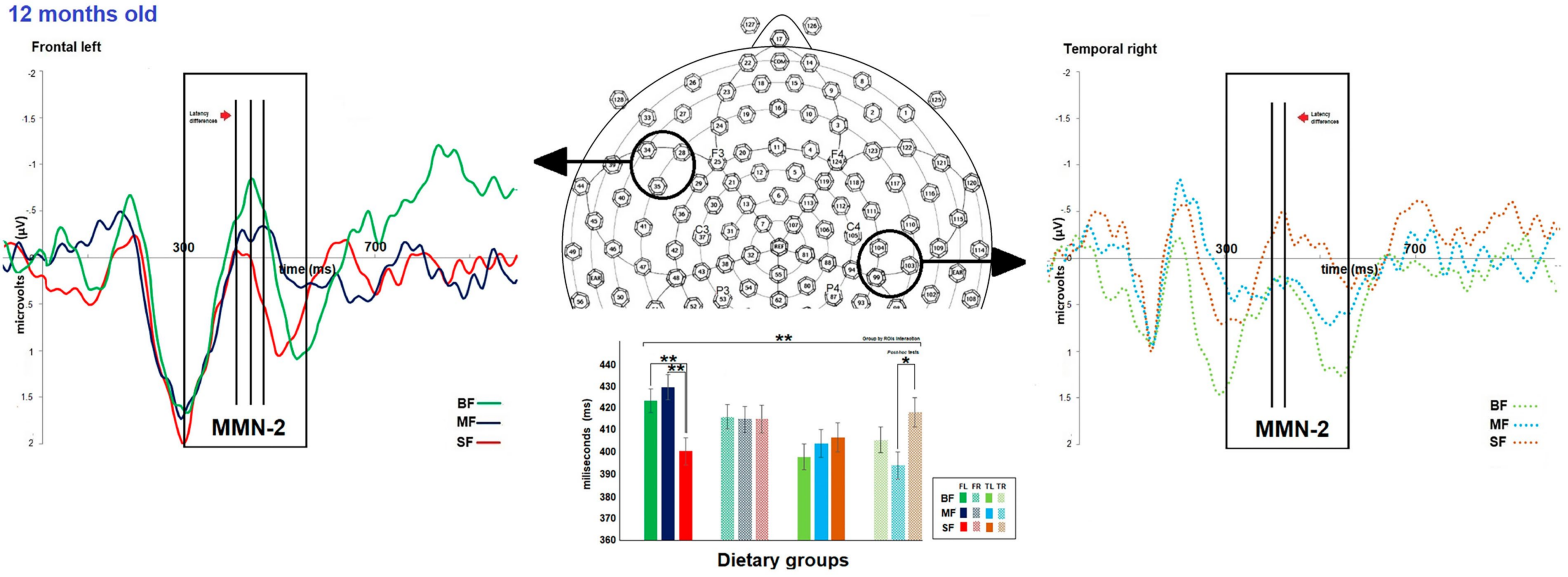
Differences between dietary groups in MMN-2 latency at 12-months old. On top, the grand average of difference wave of event-related potentials (ERPs) in the frontal left (FL) and temporal right (TR) regions of interest (ROIs) for each dietary group at 12-months old. The bar graph shows differences in MMN-2 latency between the dietary group FL and TR ROIs on the bottom. In FL ROI, the SF group displayed shorter MMN-2 latency than the other groups, while in TR ROI, the SF group showed longer MMN-2 latency than the MF group. Significant value of *p*s has been represented as follows: *p < 0.05, **p < 0.01.

The dietary groups did not differ in MMN-2 amplitude. However, differences between dietary groups were observed in MMN-2 latency at 12 months old (see Supplementary Table S2). As shown in Figure 4, at 12 months of age a significant dietary group by ROI was found [*F*(6,981) 3.1, *p* = 0.006, Ƞ^2^ = 0.02, ε = 0.9]. The *post hoc* test showed that the SF group differed from the remaining groups in MMN-2 latency in frontal left and temporal right ROIs. The SF group displayed shorter MMN-2 latency than BF and MF groups in frontal left ROI (SF vs. BF, MD = −23.8, *p* = 0.004; SF vs. MF, MD = −27.7, *p* = 0.001), while in temporal right ROI, SF group showed longer MMN-2 latency than MF group (SF vs. MF, MD = 21.7, *p* = 0.02).

##### 3.2.2.2. Hemispheric asymmetry of amplitude or latency of ERPs

###### 3.2.2.2.1. MMN-1 component

The statistical analyses evinced no differences between dietary groups in hemispheric asymmetry of MMN-1 component.

###### 3.2.2.2.2. MMN-2 component

**Figure 5:**
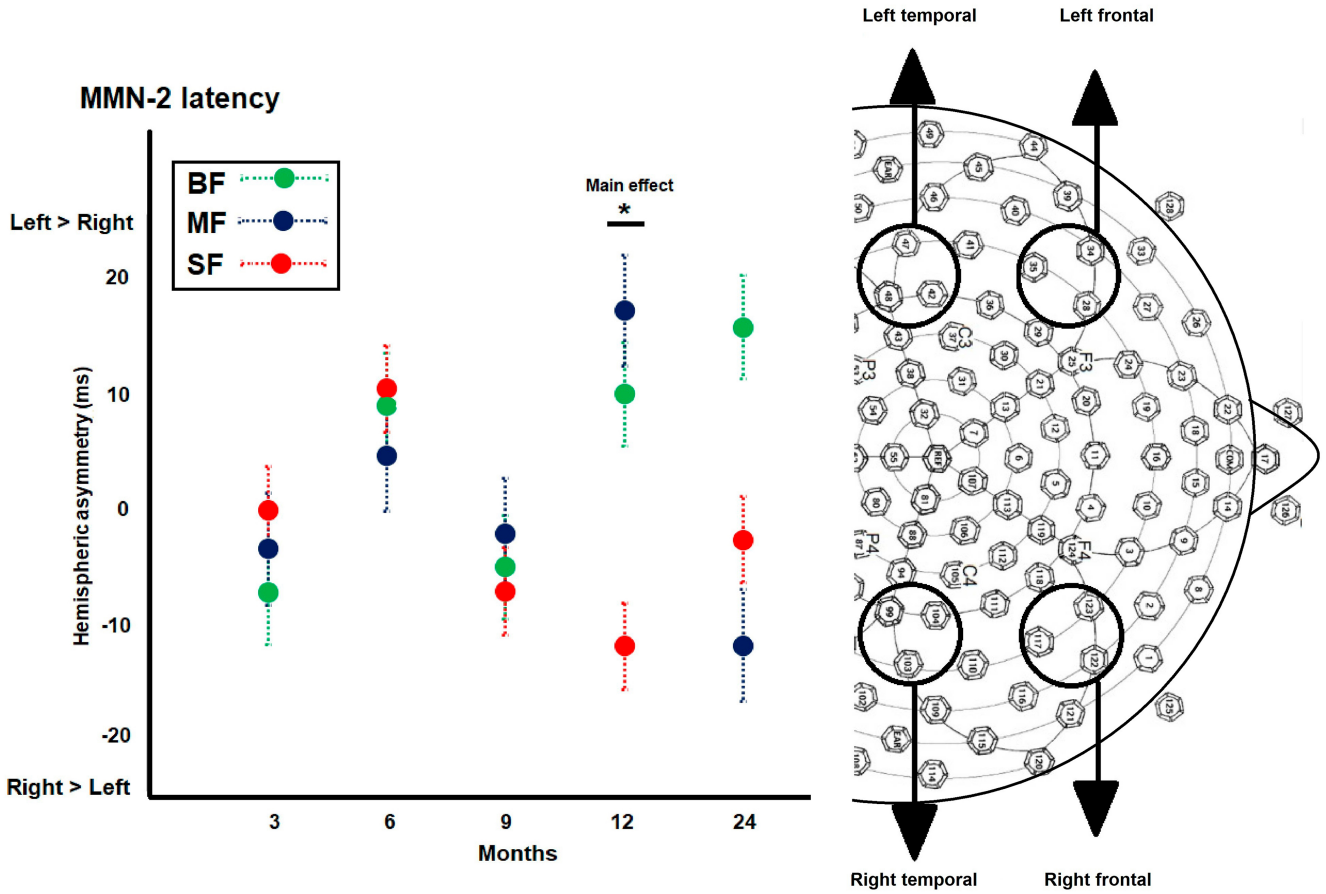
Differences between dietary groups in hemispheric asymmetry of event-related potentials (ERPs) components. The scatter plot illustrates differences between dietary groups in the hemispheric asymmetry of MMN-2 latency, which were observed at 12-months of age. Significant value of *p*s has been represented as follows: *p < 0.05.

Although the weight groups did not differ in hemispheric asymmetry of MMN-1 amplitude, they differed in MMN-2 latency at 12 months old (see Supplementary Table S3). The *post hoc* test showed significant differences between MF and SF groups (MD = 27.19, *p* = 0.002; MF, *M* = 13.72; SF, *M* = −13.56; see Figure 5). The MF infants displayed greater MMN-2 latency in left than right hemisphere, while SF group displayed the inverse pattern (MF: left, *M* = 416.78 ms, right, *M* = 404.59 ms; SF: left, *M* = 403.68 ms, right, *M* = 416.64 ms). No significant dietary group by hemispheric asymmetry of ERP component was observed.

Comparisons between age groups for each dietary group.

##### 3.2.2.3. Amplitude and latency of ERPs

###### 3.2.2.3.1. BF group

####### 3.2.2.3.1.1. MMN-1 component

Although age groups did not differ in MMN-1 amplitude, they did differ in MMN-1 latency, where a significant age group by ROI interaction was observed [*F*(12,1792) 2.08, *p* = 0.02, *Ƞ^2^* = 0.01, ε = 0.96]. The *post hoc* test evinced differences between age groups in frontal right and temporal right ROIs. In frontal right ROI, the 3-month-old infants displayed shorter MMN-1 latency than 12-months-old participants, while in temporal right ROI, infants at 24 months of age had shorter MMN-1 latency than those participants at 3, 6, and 9 months old (see Supplementary Figure S1).

####### 3.2.2.3.1.2. MMN-2 component

The age groups differed in both amplitude and latency of the MMN-2 component. A main effect of age was observed in MMN-2 amplitude [*F*(4,623) 3.29, *p* = 0.01, *Ƞ^2^* = 0.02, ε = 0.92]. The *post hoc* test revealed smaller MMN-2 amplitude in infants at 3 compared to 24 months old. The 6-month-old infants also displayed smaller amplitude than the participants at 9, 12, and 24 months old (see Supplementary Table S4).

A significant age group by ROIs interaction was also seen [*F*(12,1719) 2.86, *p* = 0.001, *Ƞ^2^* = 0.02, ε = 0.92]. The *post hoc* tests showed that age groups were different in frontal left, frontal right, and temporal right ROIs. In both frontal left and right ROIs, 3-month-old infants displayed smaller MMN-2 amplitude than infants at 12 and 24 months. We also observed that 6-month-old participants showed smaller MMN-2 amplitude than participants at 9, 12, and 24 months old in both left and right frontal ROIs, while in temporal right ROI, a greater MMN-2 amplitude was observed in 3-month-old infants compared to participants at 24 months.

The differences between age groups in MMN-2 latency were observed regardless of ROI, a main effect of age group [*F*(4,623) 3.87, *p* = 0.004, *Ƞ^2^* = 0.02, ε = 0.94] showed longer MMN-2 latency for 6-month than 3 months old participants. The 24-month-old infants also showed shorter MMN-2 latency than participants at 6, 9, and 12 months old. A significant age group by ROI interaction was also seen [*F*(12,1759) 1.86, *p* = 0.04, *Ƞ^2^* = 0.01, ε = 0.94]. The age groups differed in MMN-2 latency in frontal left, frontal right, temporal right ROIs. The *post hoc* tests showed that infants at 3 months of age displayed longer MMN-2 latency than participants at 6, 9, 12, and 24 months in frontal left ROI, while in frontal right, 3-month-old infants displayed longer MMN-2 latency than infants at 9 months of age, and the participants at 9 months of age had longer MMN-2 latency compared to 24-month-old infants. In temporal right ROI, infants at 24 months of age displayed longer MMN-2 latency than participants at 3 and 6 months old (see Supplementary Figure S1).

###### 3.2.2.3.2. MF group

####### 3.2.2.3.2.1. MMN-1 component

The age groups differed in amplitude and latency of MMN-1 component in the MF group. A significant age group by ROI interaction was seen for MMN-1 amplitude [*F*(12,1,588) 2.39, *p* = 0.007, *Ƞ^2^* = 0.02, ε = 0.89]. The *post hoc* tests showed that age groups differed in MMN-1 amplitude all ROIs (i.e., frontal left, frontal right, temporal left, and temporal right). In the frontal left ROI, infants at 3 months of age displayed smaller MMN-1 amplitude than 24-month-old participants, while in frontal right ROI, infants at 6 months of age displayed smaller MMN-1 amplitude compared to 9-and 12-month-old infants (See Supplementary Figure S2). In temporal left ROI, a smaller MMN-1 amplitude was observed in 3-month-old infants compared to 6-month-old participants. In addition, participants at 9 and 12 months of age displayed a smaller MMN-1 amplitude than 6-monts-old infants. In temporal right ROI, infants at 24 months of age displayed a smaller MMN-1 amplitude than those infants at 3 and 9 months old (see Supplementary Table S4).

In the comparisons between age groups in MMN-1 latency, we also found a significant age group by ROIs interaction [*F*(12,1705) 2.05, *p* = 0.02, *Ƞ^2^* = 0.01, ε = 0.96]. The *post hoc* tests evidenced differences between groups in frontal left, frontal right, and temporal right ROIs. In frontal left ROI, shorter MMN-1 latency was seen in infants at 3 months of age compared to the participants at 6 and 12 months old, while in frontal right a similar pattern was observed, infants at 3 months of age displayed shorter MMN-1 latency than 24-month-old infants. In temporal right ROI, the 9-month-old infants displayed longer MMN-1 latency than infants at 3 months old, and shorter MMN-1 latency compared to 24-month-old infants.

####### 3.2.2.3.2.2. MMN-2 component

Although the age groups did not differ in MMN-2 amplitude, they were different in MMN-2 latency. A significant age group by ROI interaction [*F*(12,1,667) 2.93, *p* = 0.001, *Ƞ^2^* = 0.02, ε = 0.94] revealed that the groups differed in frontal left, frontal right, and temporal left ROIs. In frontal left ROI, infants at 3 months of age displayed shorter MMN-2 latency than participants at 9 and 12 months. The participants at 6 months of age also showed shorter MMN-2 latency compared to 12-month-old infants. However, at 24 months old, the infants displayed a shorter MMN-2 latency than the participants at 9 and 12 months. In frontal right ROI, longer MMN-2 latency was seen in 9-month-old infants compared to infants at 3 and 6 months old. In temporal left ROI, we found that infants at 6 months old displayed longer MMN-2 latency than 9-month-old infants (see Supplementary Figure S2).

###### 3.2.2.3.3. SF group

####### 3.2.2.3.3.1. MMN-1 component

There were no differences between age groups in amplitude or latency of MMN-1 component.

####### 3.2.2.3.3.2. MMN-2 component

The age groups did not differ in MMN-2 amplitude, but they differed in MMN-2 latency. A significant main effect of group [*F*(4,567) 2.57, *p* = 0.04, *Ƞ^2^* = 0.02, ε = 0.96] evinced that infants at 24 months of age displayed shorter MMN-2 latency compared to infants at 6, 9, and 12 months of age (see Supplementary Table S4).

A significant age group by ROI interaction was also seen [*F*(12,1,635) 3.83, *p* < 0.001, *Ƞ^2^* = 0.03, ε = 0.96]. The *post hoc* tests showed that age groups differed in MMN-2 latency in frontal left, frontal right, temporal left, and temporal right ROIs. In frontal left ROI, infants at 3 months of age displayed shorter MMN-2 latency than participants at 6 and 9 months old. 12-months-old infants displayed shorter MMN-2 latency than participants at 6 months old. This same pattern was observed for 24-month-old infants, which displayed shorter MMN-2 latency compared to infants at 6 and 9 months old. In frontal right ROI, infants at 3 months of age also showed shorter MMN-2 latency than infants at 6, 9, and 12 months of age. However, at 24 months, infants displayed shorter MMN-2 latency than 9-month-old infants. In the temporal left ROI, the participants at 3 months of age displayed longer MMN-2 latency than 9-month-old infants, while in temporal right ROI, 3-month-old participants displayed longer MMN-2 latency compared to infants at 24 months of age and infants at 12 months old displayed longer MMN-2 latency than participants at 6 and 24 months old (see Supplementary Figure S3).

#### 3.2.3. Regression results

As shown in Table 3, at 12-months old, only the infant’s diet predicted MMN-2 latency in frontal left and temporal right ROIs. In this same age group, diet was also a predictor of hemispheric asymmetry in the MMN-2 latency.

**Table 3:** Regression models predicting latency or hemispheric asymmetry of MMN-2 component at 12-months old.

Age		Variables		Coefficient standardized			Model	ANOVA	
		ROI	Predictor	*β*	*t*	*p*-value	*R* ^2^	*F*	*p*-value
MMN-2 latency									
12 m	FL	Diet	0.1	2.8	0.005	0.02	8.0	0.005**
	TR	Diet	−0.1	−2.0	0.05	0.01	3.9	0.05*	Hemispheric asymmetry of MMN-2 latency
12 m	Frontal	Diet	0.1	2.2	0.02	0.01	5.0	0.02*

m, months; FL, frontal left; FR, frontal right; TR, temporal right. *p < 0.05; **p < 0.01.

## 4. Discussion

This study sought to identify electrophysiological differences between dietary groups at 3, 6, 9, 12, and 24 months of age. We expected to find an effect of diet on infant phonological processing, particularly at earlier developmental ages, which would be characterized by greater amplitude and shorter latency of MMN components and accompanied by a greater hemispheric asymmetry of MMN components for the BF group than MF and SF groups. Additionally, we expected greater amplitude and shorter latencies of MMN components for BF groups as age increased, which we expected to be less evident in the other dietary groups.

### 4.1. Phonological-perception development between dietary groups

Our findings partially matched our hypothesis with differences between dietary groups observed in only one of the MMN components. We did not find differences between dietary groups in amplitude or latency of MMN-1, which has been associated with the identification of acoustic features of a stimulus (i.e., MMN-1) (51–53). Although this finding is in line with findings of Li et al. (13), it did not match those reported by Pivik et al. (3) who reported differences in P1 amplitude between dietary groups, with greater amplitude in the deviant condition for soy milk fed infants than breastfed at 3 and 6-months. In our study we expected to find a greater MMN-1 amplitude for SF than BF groups, in keeping with the findings of Pivik et al. (3), but this was not the case. We suggest that our results could be explained by the type of ERP analyses performed. Pivik et al. (3) compared the amplitude and latency of the P1 component associated with frequent and deviant conditions, while in our study, we directly compared the differences between experimental conditions (i.e., MMN components), and included infant sex and gestation weeks as covariates.

We propose that our results might be explained by the suggestion of previous studies that identification of acoustic features is developed very early in infancy (30–32). Given that this precognitive process might not be under development during our evaluation period, the nutritional requirements to support brain networks need for efficient processing would be easily provided by each of the three diets evaluated.

We also hypothesized differences between dietary groups in the MMN-2 component at six and 12-months of age. Our results partially supported our hypotheses; dietary groups only differed at 12-months old, underpinning the idea that nutrient intake has a greater effect on an infant’s cognition at a critical stage of language development. At this age, it is expected that infants show phonemic normalization and categorical perception (30]. Infants should recognize words (36) because they have already undergone extensive maturity changes in brain networks associated with production centers in the frontal region and the phonological store in the temporal region (38). Moreover, they already show a more mature hemispheric specialization associated with language processing (40, 77). As a consequence, phonological perception might require greater participation from neural networks that support attentional monitoring, inhibitory control, stimulus detection, and working memory (i.e., the dorsolateral prefrontal cortex, inferior frontal junction, inferior frontal gyrus, insula, presupplementary motor area, subthalamic nucleus, median cingulate, and striatum) (78) because they have attended syllables and inhibit their possible meaning in their native language, promoting greater participation from frontal brain areas related to attention-inhibition processing (38).

Our findings revealed that the SF group showed an inverse electrophysiological pattern to that of BF and MF infants; in which the SF group displayed shorter MMN-2 latency in frontal left ROI and longer MMN-2 latency in temporal right ROI. One explanation for the differences between dietary groups in MMN-2 latency in frontal left ROI is that the SF group exhibits a different attention-inhibition effort than the other groups, reflected in a reduced level of interference relative to the other groups. While shorter latencies might suggest more efficient processing, this finding might also indicate that SF infants have less linguistic information to inhibit or a weaker attention-inhibition brain network. This last explanation matches the findings of Li et al. (79) who reported lower executive function in children fed with soy formula in infancy than those fed with breast or cow-milk formula.

On the other hand, shorter MMN-2 latency for the SF group in temporal right ROI requires an additional explanation. Although how the hemispheric specialization in language processing develops during infancy is still debated, it has been hypothesized that the left hemisphere is specialized for speech stimuli, while the right hemisphere supports the auditory identification of non-speech stimuli (80, 81). In our study, the SF group displayed an enhanced response in the right hemisphere, suggesting that this group is attending the syllables as non-speech stimuli. This proposal is in accord with their brain response in frontal left ROI. They appear to expend less cognitive effort to attend syllables and inhibit linguistic context because they may be processing the syllables as tones (81). The SF group also exhibited a more right-lateralized MMN-2 asymmetry that has been suggested to be associated with a risk of delayed language development (40). Attenuation of left hemispheric ERPs (82, 83) or atypical enhanced responses in the right hemisphere (84, 85) have been to confer greater risk of poor language development. Moreover, given that regression analyses indicated that only infant diet predicted latency and hemispheric asymmetry of the MMN-2 component in frontal areas, the SF group’s electrophysiological response might indicate a deviation from normal language development after a prolonged use of soy-based formula.

In addition, the electrophysiological pattern observed in SF groups does not match the temporal gradient in information processing (i.e., faster processing in temporal than frontal regions) observed in normal development (54). These findings addressed the speed at which information is processed between language areas, suggesting that the differences between dietary groups in frontal ROIs could be interpreted as modulations in brain networks to enhance the ability to distinguish between syllables and manage neural resources and cognitive effort.

Prior studies using animal models and humans have noted that soy food contains phytoestrogens such as isoflavones (86–88) that seem to have a negative effect on cognition, alter sexually dimorphic brain regions, learning, memory (89) and executive functions (79). We suggest that the deviation from normal language processing observed in the SF group may be attributable to the composition of soy formulas.

### 4.2. Phonological-perception development for each dietary group

Consistent with our hypothesis, dietary groups displayed changes in MMN components associated with age, and these changes were more evident in the BF group. The MMN-1 component appears to change with age only in the BF and MF groups. Both groups displayed an increase in MMN-1 latency in frontal ROIs, which may suggest greater participation of frontal areas supporting inhibitory control in order to better identify the features of acoustic stimuli (51–53). These dietary groups also displayed a decrease in MMN-latency in temporal ROIs, which might be explained as a reflection of a more available phonological store (38) as age increases. However, SF infants did not display these changes associated with age, suggestive of a less stable development of the ability to identify the features of acoustic stimuli. Another explanation for this result is that the SF group had high variability in their brain-electrical responses associated with identification of acoustic features at all ages, which would hamper the observation of differences between age groups and even more so between dietary groups.

Although the MMN-2 component changed with age in all dietary groups, only the BF group showed greater MMN-2 amplitude as age increased, as has been described in a previous study (50). This finding may be interpreted as greater availability of neural resources in older breastfed infants who seemed to show a greater stimulus awareness and perceptual salience, and thus a greater index of auditory recognition memory (51, 90) as age increased. This finding is consistent with behavioral results observed in 24-month-old infants on the PLS-3 test where breastfed infants showed greater auditory comprehension.

On the other hand, the electrophysiological pattern associated with age of MMN-2 latency also depended on regions of interest. BF and MF groups showed an increase in MMN-2 latency in the frontal left ROI from 3 to 12 months of age. This pattern was not observed in the SF group. Instead, that group displayed a concave-learning curve (91) characterized by a significant decrease of MMN-2 latency in frontal left ROI from 12 months of age. This finding may indicate reduced participation of the frontal left ROI in auditory recognition memory, consistent with a deviation from normal development in the recruitment of this brain area to process phonological awareness. In addition, an unexpected result was that SF infants displayed an increase in MMN-2 latency in temporal regions from this same age, which contrasted with the decreased age-associated finding in BF and MF groups. A previous study of language learning has described those greater fluctuations in learning curves as an indicator of slower learners, which may explain our findings in SF group (91). We add to this that SF infants may have a less available phonological store at 12 months of age. The unexpected electrophysiological pattern observed in SF infants temporally matches with a milestone in infant language development where they are expected to show greater stimulus awareness due to their ability to distinguish words, syllables, and tones. Therefore, we suggest that SF infants compensate for failures in the frontal left area by recruiting bilateral temporal ROI to distinguish between phonological features of words, syllables, and tones.

### 4.3. Limitations

There are inherent limitations in the present study. Although the longitudinal nature of this study may support interpretations of causality between diet and phonological processing, it is essential to highlight that the same subjects did not always constitute the sample at each moment evaluated. Some of them missed more than one measurement. Therefore, interpretations should be carried out carefully. In addition, given that our study implied infant nutrition, variables surrounding infant feeding were not wholly controlled, among them the mother’s health or the amount of food provided to the infant, or complementary feeding habits. We did not explore why the parents choose one of the three diets offered. In this study, we used traditional anthropometric measures to assess the participants, while this is a common use of body composition measures (e.g., energy X-ray absorptiometry) or biochemical indices, these might provide more nuanced metrics for studies examining the impact of diet on neural maturation and cognitive function in infants.

## Data availability statement

The raw data supporting the conclusions of this article will be made available by the authors, without undue reservation.

## Ethics statement

The studies involving human participants were reviewed and approved by Institutional Review Board of the University of Arkansas for Medical Sciences. Written informed consent to participate in this study was provided by the participants’ legal guardian/next of kin.

## Author contributions

GA-C, AA, SS, and LL-P contributed to the conception and design of the study. YG, DW, HD, and DH organized the database for the statistical analyses. All authors contributed to the article and approved the submitted version.

## Funding

This research was supported by USDA/Agricultural Research Service Project 6026-51000-012-06S.

## Conflict of interest

The authors declare that the research was conducted in the absence of any commercial or financial relationships that could be construed as a potential conflict of interest.

## Publisher’s note

All claims expressed in this article are solely those of the authors and do not necessarily represent those of their affiliated organizations, or those of the publisher, the editors and the reviewers. Any product that may be evaluated in this article, or claim that may be made by its manufacturer, is not guaranteed or endorsed by the publisher.
