# Supplementary material for: Impact of feeding habits on the development of language-specific processing of phonemes in brain: An event-related potentials study
Source: Front Nutr. 2023 Feb 17;10:1032413. doi: 10.3389/fnut.2023.1032413 (PMC9982124; doi:10.3389/fnut.2023.1032413)
Supplement: Supplementary file 1 [file Table_1.docx]

**Table 1.** Amplitude and latency of MMN-1 by dietary group

|  |  | **Dietary group** | | | **Main effect of group** | | |
| --- | --- | --- | --- | --- | --- | --- | --- |
| **Age** | **ROIs** | **BF** | **MF**  **Mean (SD)** | **SF** | **F** | ***p*** | ***Ƞ^2^*** |
| **Amplitude analyses “µV”** | | | | | | | |
| 3 m | Frontal left | .2 (2.5) | .6 (2.4) | .4 (2.8) | F (2, 400) .05 | .9 | .000 |
|  | Frontal right | .5 (2.7) | .1 (2.7) | .2 (2.6) |  |  |  |
|  | Temporal left | -.1 (2.9) | .1 (2.4) | -.02 (3.1) |  |  |  |
|  | Temporal right | .1 (2.7) | -.2 (2.7) | .2 (2.4) |  |  |  |
| 6 m | Frontal left | .3 (2.9) | .4 (3.4) | .5 (3.5) | F (2, 358) .4 | .7 | .002 |
|  | Frontal right | .3 (2.9) | .7 (2.9) | .5 (3.1) |  |  |  |
|  | Temporal left | .2 (2.7) | -.7 (2.8) | -.05 (2.8) |  |  |  |
|  | Temporal right | .05 (3.0) | .1 (3.2) | .1 (2.9) |  |  |  |
| 9 m | Frontal left | .3 (3.2) | .1 (3.1) | .03 (3.0) | F (2, 330) .3 | .8 | .002 |
|  | Frontal right | .002 (3.9) | -.2 (3.1) | -.03 (2.8) |  |  |  |
|  | Temporal left | -.3 (2.9) | .1 (2.4) | -.4 (2.6) |  |  |  |
|  | Temporal right | .09 (3.2) | -.4 (2.7) | .2 (2.2) |  |  |  |
| 12 m | Frontal left | .05 (3.6) | -.01 (3.1) | -.3 (3.1) | F (2, 326) .5 | .6 | .003 |
|  | Frontal right | -.2 (2.9) | -.1 (3.2) | .2 (3.1) |  |  |  |
|  | Temporal left | -.2 (2.7) | -.07 (2.8) | -.09 (2.7) |  |  |  |
|  | Temporal right | .4 (2.7) | -.04 (2.4) | -.01 (2.4) |  |  |  |
| 24 m | Frontal left | -.06 (2.6) | -.3 (2.6) | .1 (2.6) | F (2, 365) 1.3 | .3 | .007 |
|  | Frontal right | -.2 (2.5) | .1 (2.6) | .1 (2.6) |  |  |  |
|  | Temporal left | .2 (1.9) | -.2 (2.2) | .2 (2.1) |  |  |  |
|  | Temporal right | .2 (2.0) | .2 (1.8) | .4 (2.2) |  |  |  |
|  |  | **Latency analyses “ms”** | | |  |  |  |
| 3 m | Frontal left | 156.0 (61.9) | 152.9 (62.2) | 159.8 (62.8) | F (2, 403) 1.2 | .3 | .006 |
|  | Frontal right | 156.2 (60.5) | 158.9 (62.6) | 152.1 (61.9) |  |  |  |
|  | Temporal left | 168.4 (64.3) | 173.3 (61.3) | 176.6 (61.4) |  |  |  |
|  | Temporal right | 178.2 (67.2) | 162.4 (64.3) | 183.0 (65.7) |  |  |  |
| 6 m | Frontal left | 161.1 (57.6) | 167.8 (62.9) | 157.9 (61.8) | F (2, 355) .3 | .7 | .002 |
|  | Frontal right | 168.6 (52.7) | 164.6 (57.0) | 158.8 (62.7) |  |  |  |
|  | Temporal left | 168.1 (59.4) | 169.4 (58.0) | 170.1 (58.5) |  |  |  |
|  | Temporal right | 177.6 (61.9) | 170.7 (64.4) | 175.7 (64.1) |  |  |  |
| 9 m | Frontal left | 166.6 (59.7) | 159.2 (59.1) | 163.0 (55.7) | F (2, 331) .8 | .4 | .005 |
|  | Frontal right | 159.5 (55.2) | 167.7 (55.6) | 163.2 (50.4) |  |  |  |
|  | Temporal left | 162.8 (59.4) | 171.5 (60.7) | 171.3 (60.5) |  |  |  |
|  | Temporal right | 175.6 (56.1) | 184.8 (57.0) | 169.5 (62.7) |  |  |  |
| 12 m | Frontal left | 160.4 (57.3) | 167.2 (58.7) | 164.2 (58.0) | F (2, 327) .1 | .8 | .000 |
|  | Frontal right | 169.5 (52.0) | 168.6 (53.0) | 158.9 (55.2) |  |  |  |
|  | Temporal left | 174.5 (56.3) | 160.7 (60.0) | 170.8 (58.4) |  |  |  |
|  | Temporal right | 167.5 (54.1) | 171.7 (62.7) | 175.1 (58.9) |  |  |  |
| 24 m | Frontal left | 167.6 (56.5) | 172.3 (59.6) | 165.1 (60.3) | F (2, 363) .8 | .4 | .004 |
|  | Frontal right | 165.0 (56.7) | 174.3 (58.3) | 168.4 (60.2) |  |  |  |
|  | Temporal left | 162.4 (58.0) | 164.4 (60.0) | 164.0 (60.7) |  |  |  |
|  | Temporal right | 154.8 (61.0) | 156.5 (58.6) | 163.0 (61.8) |  |  |  |

m = months; ROIs = Regions of interest; BF = Breast fed; MF = Milk fed; SF = Soy fed; SD = standard deviation
