# Supplementary material for: Impact of feeding habits on the development of language-specific processing of phonemes in brain: An event-related potentials study
Source: Front Nutr. 2023 Feb 17;10:1032413. doi: 10.3389/fnut.2023.1032413 (PMC9982124; doi:10.3389/fnut.2023.1032413)
Supplement: Supplementary file 2 [file Table_2.docx]

**Table 2.** MMN-2 amplitude by dietary group

|  |  | **Dietary group** | | | **Main effect of group** | | |
| --- | --- | --- | --- | --- | --- | --- | --- |
| **Age** | **ROIs** | **BF** | **MF**  **Mean (SD)** | **SF** | **F** | ***p*** | ***Ƞ^2^*** |
|  |  | **Amplitude analyses “µV”** | | |  |  |  |
| 3 m | Frontal left | 1.4 (3.3) | 1.1 (3.7) | 1.1 (3.4) | F (2, 400) .09 | .9 | .000 |
|  | Frontal right | 1.3 (4.0) | .7 (3.6) | .9 (4.0) |  |  |  |
|  | Temporal left | .2 (3.9) | .7 (3.5) | .4 (3.4) |  |  |  |
|  | Temporal right | -.1 (3.3) | .5 (3.2) | .3 (3.3) |  |  |  |
| 6 m | Frontal left | 1.5 (4.3) | .6 (4.1) | .8 (4.3) | F (2, 358) 2.6 | .08 | .01 |
|  | Frontal right | 1.4 (3.8) | 1.0 (3.8) | 1.0 (3.7) |  |  |  |
|  | Temporal left | .5 (3.6) | -.3 (3.2) | .3 (3.5) |  |  |  |
|  | Temporal right | .1 (3.8) | .04 (3.9) | .3 (4.4) |  |  |  |
| 9 m | Frontal left | .4 (4.4) | .9 (4.0) | .9 (4.4) | F (2, 330) .5 | .6 | .003 |
|  | Frontal right | .3 (4.3) | .3 (3.5) | 1.1 (3.6) |  |  |  |
|  | Temporal left | .06 (3.8) | .6 (3.2) | -.4 (3.2) |  |  |  |
|  | Temporal right | .4 (3.9) | -.05 (3.2) | .8 (3.3) |  |  |  |
| 12 m | Frontal left | .2 (4.4) | .4 (4.1) | .7 (3.9) | F (2, 326) .2 | .8 | .001 |
|  | Frontal right | -.03 (4.6) | .7 (4.2) | .9 (4.5) |  |  |  |
|  | Temporal left | .4 (3.6) | .1 (3.4) | .2 (3.5) |  |  |  |
|  | Temporal right | .6 (3.5) | .3 (3.5) | .03 (3.0) |  |  |  |
| 24 m | Frontal left | -.1 (3.6) | .2 (3.3) | .5 (3.8) | F (2, 365) .3 | .7 | .002 |
|  | Frontal right | -.2 (3.4) | .1 (3.2) | -.04 (3.7) |  |  |  |
|  | Temporal left | .4 (2.6) | .3 (2.7) | .5 (2.8) |  |  |  |
|  | Temporal right | .8 (2.4) | .5 (2.5) | .6 (2.9) |  |  |  |
|  |  | **Latency analyses “ms”** | | |  |  |  |
| 3 m | Frontal left | 398.6 (59.3) | 397.0 (61.4) | 395.0 (56.7) | F (2, 403) .09 | .9 | .000 |
|  | Frontal right | 409.1 (57.5) | 403.2 (53.6) | 397.8 (56.0) |  |  |  |
|  | Temporal left | 410.0 (74.1) | 408.3 (71.3) | 413.0 (70.5) |  |  |  |
|  | Temporal right | 407.0 (69.1) | 411.6 (73.7) | 413.4 (71.1) |  |  |  |
| 6 m | Frontal left | 422.5 (61.8) | 412.0 (69.8) | 422.1 (62.6) | F (2, 355) 2.6 | .07 | .01 |
|  | Frontal right | 416.3 (64.0) | 408.6 (63.4) | 415.3 (63.3) |  |  |  |
|  | Temporal left | 409.0 (70.8) | 414.2 (68.1) | 399.2 (70.6) |  |  |  |
|  | Temporal right | 415.4 (63.9) | 400.0 (67.6) | 399.2 (65.9) |  |  |  |
| 9 m | Frontal left | 417.0 (63.7) | 421.8 (69.5) | 416.2 (66.0) | F (2, 331) .01 | 1.0 | .000 |
|  | Frontal right | 424.6 (55.8) | 426.0 (65.0) | 426.1 (60.6) |  |  |  |
|  | Temporal left | 399.5 (65.9) | 394.5 (70.6) | 390.0 (70.0) |  |  |  |
|  | Temporal right | 400.1 (62.0) | 396.4 (67.7) | 403.1 (66.5) |  |  |  |
| 12 m | Frontal left | 423.4 (58.6) | 428.9 (62.4) | 400.4 (60.9) | F (2, 327) .08 | .9 | .001 |
|  | Frontal right | 416.2 (59.6) | 415.6 (66.4) | 415.4 (59.3) |  |  |  |
|  | Temporal left | 398.0 (67.2) | 404.2 (64.8) | 406.2 (65.2) |  |  |  |
|  | Temporal right | 405.7 (62.9) | 395.0 (69.3) | 417.3 (63.1) |  |  |  |
| 24 m | Frontal left | 416.1 (59.2) | 404.2 (63.2) | 396.8 (66.2) | F (2, 363) 2.4 | .1 | .01 |
|  | Frontal right | 403.3 (58.4) | 418.1 (62.7) | 401.7 (65.2) |  |  |  |
|  | Temporal left | 394.5 (63.4) | 401.5 (64.8) | 406.0 (65.9) |  |  |  |
|  | Temporal right | 391.2 (61.5) | 403.0 (62.0) | 387.0 (61.7) |  |  |  |

m = months; ROIs = Regions of interest; BF = Breast fed; SF = Soy fed; MF = Milk fed; SD = standard deviation.
