# Supplementary material for: Impact of feeding habits on the development of language-specific processing of phonemes in brain: An event-related potentials study
Source: Front Nutr. 2023 Feb 17;10:1032413. doi: 10.3389/fnut.2023.1032413 (PMC9982124; doi:10.3389/fnut.2023.1032413)
Supplement: Supplementary file 3 [file Table_3.docx]

**Table 3. Hemispheric asymmetry of amplitude and latency of ERP components**

|  |  | **Main effect of group** | | |
| --- | --- | --- | --- | --- |
| **Age** | **ERP component** | **F** | ***p*** | ***Ƞ^2^*** |
| **Amplitude analyses** | | | | |
| 3 m | MMN-1 | F(2,400) 1.35 | .26 | .007 |
|  | MMN-2 | ‘’ .05 | .95 | .000 |
| 6 m | MMN-1 | F(2,358) 1.28 | .28 | .007 |
|  | MMN-2 | ‘’ .57 | .57 | .003 |
| 9 m | MMN-1 | F(2,330) 1.71 | .18 | .01 |
|  | MMN-2 | ‘’ 2.80 | .06 | .02 |
| 12 m | MMN-1 | F(2,326) .11 | .90 | .002 |
|  | MMN-2 | ‘’ .19 | .83 | .001 |
| 24 m | MMN-1 | F(2, 365) 2.16 | .12 | .01 |
|  | MMN-2 | ‘’ .16 | .85 | .001 |
| **Latency analyses** | | | | |
| 3 m | MMN-1 | F(2,400) .93 | .40 | .005 |
|  | MMN-2 | ‘’ .07 | .94 | .000 |
| 6 m | MMN-1 | F(2,358) .31 | .73 | .002 |
|  | MMN-2 | ‘’ .36 | .70 | .002 |
| 9 m | MMN-1 | F(2,330) 1.46 | .23 | .009 |
|  | MMN-2 | ‘’ .84 | .43 | .005 |
| 12 m | MMN-1 | F(2,326) .49 | .61 | .003 |
|  | MMN-2 | ‘’ 4.9 | .008** | .03 |
| 24 m | MMN-1 | F(2,365) .33 | .72 | .002 |
|  | MMN-2 | ‘’ 2.24 | .11 | .01 |

m = months; MMN = Mismatch negativity; *p < .05; **p < .01.
