# Supplementary material for: Impact of feeding habits on the development of language-specific processing of phonemes in brain: An event-related potentials study
Source: Front Nutr. 2023 Feb 17;10:1032413. doi: 10.3389/fnut.2023.1032413 (PMC9982124; doi:10.3389/fnut.2023.1032413)
Supplement: Supplementary file 4 [file Table_4.docx]

**Table 4. Mean amplitude and latency of ERP components in dietary groups**

|  |  | **BF** | | **MF** | | **SF** | |
| --- | --- | --- | --- | --- | --- | --- | --- |
| **Age** | **ROIs** | **ERP components** | | | | | |
|  |  | **MMN-1** | **MMN-2** | **MMN-1** | **MMN-2** | **MMN-1** | **MMN-2** |
| **Amplitude (µV) (SD)** | | | | | | | |
| 3 m | Frontal left | .26 (.25) | 1.40 (.34) | .55 (.25) | 1.10 (.33) | .42 (.26) | 1.10 (.34) |
|  | Frontal right | .57 (.24) | 1.30 (.34) | .16 (.25) | .69 (.32) | .23 (.25) | .88 (.34) |
|  | Temporal left | -.12 (.23) | .21 (.30) | .12 (.22) | .67 (.28) | -.03 (.23) | .42 (.28) |
|  | Temporal right | .15 (.23) | -.05 (.30) | -.17 (.23) | .54 (.29) | .23 (.21) | .32 (.30) |
| 6 m | Frontal left | .31 (.27) | 1.55 (.36) | .39 (.26) | .62 (.34) | .47 (.28) | .85 (.36) |
|  | Frontal right | .32 (.26) | 1.42 (.37) | .72 (.26) | .97 (.33) | .49 (.26) | .98 (.35) |
|  | Temporal left | .17 (.24) | .54 (.32) | -.74 (.23) | -.28 (.29) | -.05 (.25) | .27 (.30) |
|  | Temporal right | .04 (.25) | .10 (.31) | .11 (.23) | .04 (.30) | .11 (.23) | .33 (.32) |
| 9 m | Frontal left | .32 (.28) | .45 (.37) | .12 (.29) | .88 (36) | .03 (.29) | .87 (.38) |
|  | Frontal right | .003 (.27) | .32 (.38) | -.24 (.27) | .29 (.35) | -.04 (.27) | 1.10 (.37) |
|  | Temporal left | -.33 (.25) | .06 (.33) | .14 (.24) | .61 (.31) | -.41 (.26) | -.37 (.31) |
|  | Temporal right | .10 (.25) | .38 (.32) | -.42 (.25) | -.05 (.31). | .25 (.23) | .81 (.33) |
| 12 m | Frontal left | .04 (.27) | .24 (.36) | -.02 (.23) | .42 (.37) | .28 (.30) | .74 (.40) |
|  | Frontal right | -.24 (.26) | -.03 (.36) | -.10 (.27) | .72 (.35) | .22 (.29) | .94 (.39) |
|  | Temporal left | -.23 (.24) | .43 (.32) | -.07 (.24) | .09 (.31) | -.08 (.27) | .17 (.33) |
|  | Temporal right | .44 (.24) | .61 (.31) | -.04 (.25) | .35 (.31) | -.01 (.25) | .03 (.35) |
| 24 m | Frontal left | -.07 (.25) | -.13 (.33) | -.30 (.27) | .22 (.37) | .13 (.28) | .48 (.37) |
|  | Frontal right | -.19 (.24) | -.22 (.34) | .10 (.27) | .14 (.34) | .14 (.27) | -.03 (.37) |
|  | Temporal left | .22 (.22) | .46 (.29) | -.17 (.23) | .34 (.30) | .21 (.26) | .54 (.31) |
|  | Temporal right | .17 (.23) | .78 (.28) | .52 (.24) | .47 (.31) | .45 (.23) | .66 (.33) |
| **Latency (ms) (SD)** | | | | | | | |
| 3 m | Frontal left | 155.28 (5.04) | 398.28 (5.21) | 153.00 (5.15) | 397.12 (5.56) | 160.21 (5.18) | 394.98 (5.40) |
|  | Frontal right | 156.16 (4.79) | 408.44 (5.09) | 159.06 (4.91) | 403.33 (5.29) | 152.16 (5.09) | 398.07 (5.27) |
|  | Temporal left | 168.50 (5.14) | 409.62 (5.84) | 173.44 (5.10) | 408.51 (5.78) | 176.52 (5.20) | 413.45 (5.95) |
|  | Temporal right | 178.52 (5.20) | 407.24 (5.52) | 162.46 (5.25) | 411.55 (5.83) | 182.53 (5.43) | 413.58 (5.68) |
| 6 m | Frontal left | 161.84 (5.39) | 422.85 (5.57) | 167.76 (5.43) | 412.00 (5.87) | 158.33 (5.54) | 422.13 (5.78) |
|  | Frontal right | 168.63 (5.11) | 416.85 (5.44) | 164.60 (5.18) | 408.54 (5.58) | 158.84 (5.42) | 415.52 (5.63) |
|  | Temporal left | 168.03 (5.49) | 409.14 (6.23) | 169.38 (5.38) | 414.25 (6.09) | 170.01 (5.56) | 399.17 (6.36) |
|  | Temporal right | 177.26 (5.56) | 415.07 (5.89) | 170.77 (5.54) | 399.86 (6.15) | 175.28 (5.81) | 399.45 (6.08) |
| 9 m | Frontal left | 166.09 (5.54) | 416.78 (5.72) | 159.20 (5.72) | 421.76 (6.18) | 161.85 (5.69) | 415.97 (5.93) |
|  | Frontal right | 159.52 (5.25) | 424.17 (5.59) | 167.56 (5.46) | 425.66 (5.88) | 163.01 (5.57) | 425.52 (5.78) |
|  | Temporal left | 162.85 (5.65) | 399.47 (6.41) | 171.51 (5.67) | 394.33 (6.42) | 171.53 (5.71) | 390.13 (6.53) |
|  | Temporal right | 175.81 (5.71) | 400.26 (5.50) | 185.00 (5.84) | 396.54 (6.48) | 170.62 (5.96) | 402.97 (6.24) |
| 12 m | Frontal left | 159.61 (5.32) | 423.14 (5.50) | 167.16 (5.74) | 428.92 (6.21) | 164.00 (6.02) | 400.27 (6.28) |
|  | Frontal right | 169.84 (5.05) | 415.57 (5.37) | 168.77 (5.48) | 415.87 (5.91) | 158.81 (5.89) | 415.19 (6.12) |
|  | Temporal left | 174.52 (5.42) | 398.51 (6.15) | 160.69 (5.70) | 404.23 (6.45) | 170.79 (6.04) | 406.27 (6.92) |
|  | Temporal right | 168.11 (5.49) | 405.79 (5.82) | 171.53 (5.86) | 394.78 (6.51) | 175.29 (6.31) | 417.77 (6.60) |
| 24 m | Frontal left | 168.82 (4.98) | 416.54 (5.14) | 172.24 (5.63) | 404.15 (6.09) | 165.36 (5.69) | 397.08 (5.91) |
|  | Frontal right | 164.77 (4.72) | 404.23 (5.02) | 174.29 (5.38) | 418.10 (5.79) | 168.55 (5.54) | 402.01 (5.76) |
|  | Temporal left | 162.39 (5.07) | 394.25 (5.76) | 164.38 (5.59) | 401.42 (6.32) | 163.55 (5.68) | 405.95 (6.51) |
|  | Temporal right | 154.09 (5.13) | 390.96 (5.44) | 156.52 (5.75) | 403.00 (6.38) | 162.66 (5.94) | 386.20 (6.22) |

m = months; MMN = Mismatch negativity; BF = Breast fed; SF = Soy fed; MF = Milk fed; SD = standard deviation.
