# Supplementary figures and images for: Impact of feeding habits on the development of language-specific processing of phonemes in brain: An event-related potentials study
Source: Front Nutr. 2023 Feb 17;10:1032413. doi: 10.3389/fnut.2023.1032413 (PMC9982124; doi:10.3389/fnut.2023.1032413)

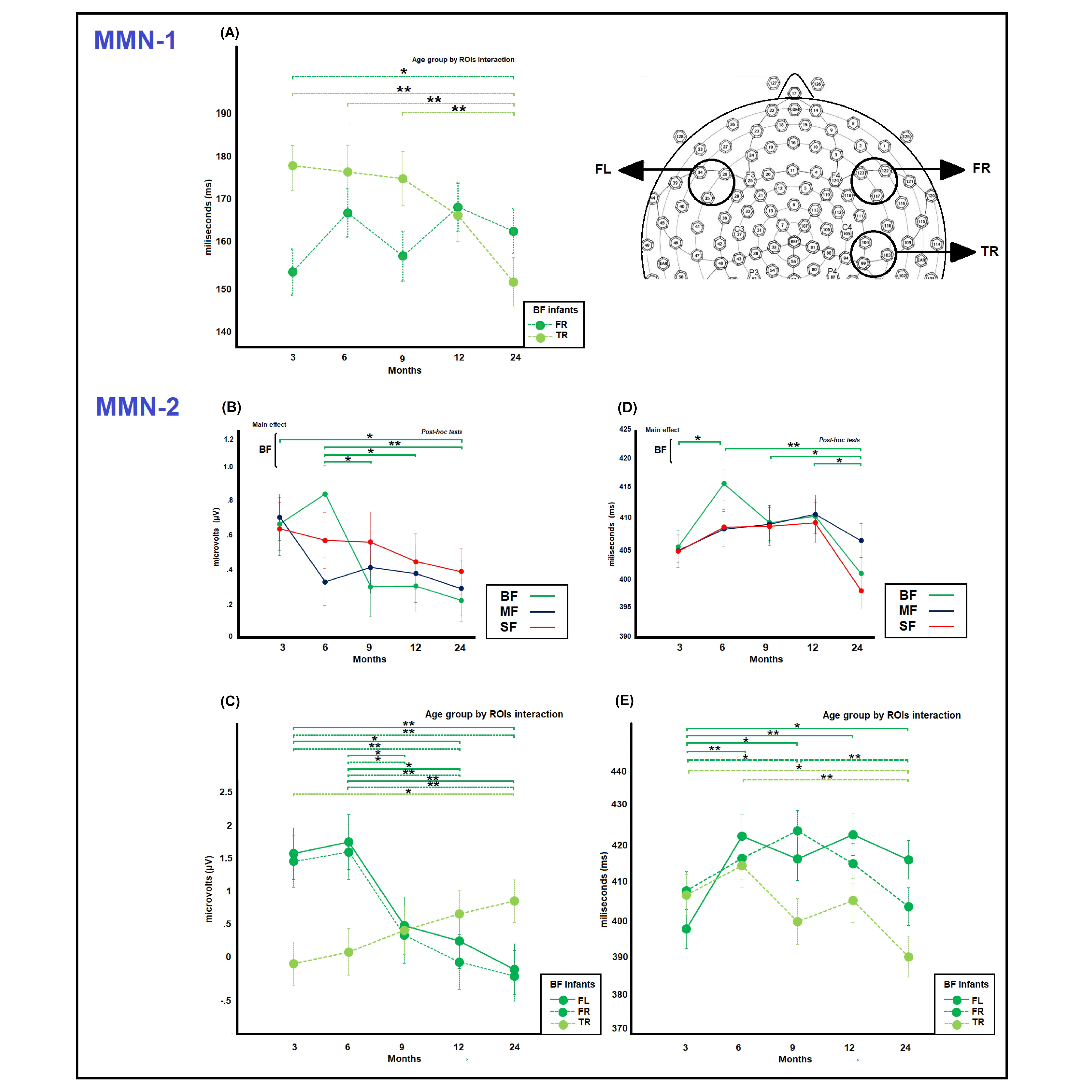

Supplement: Supplementary file 5 [file Image_1.JPEG]

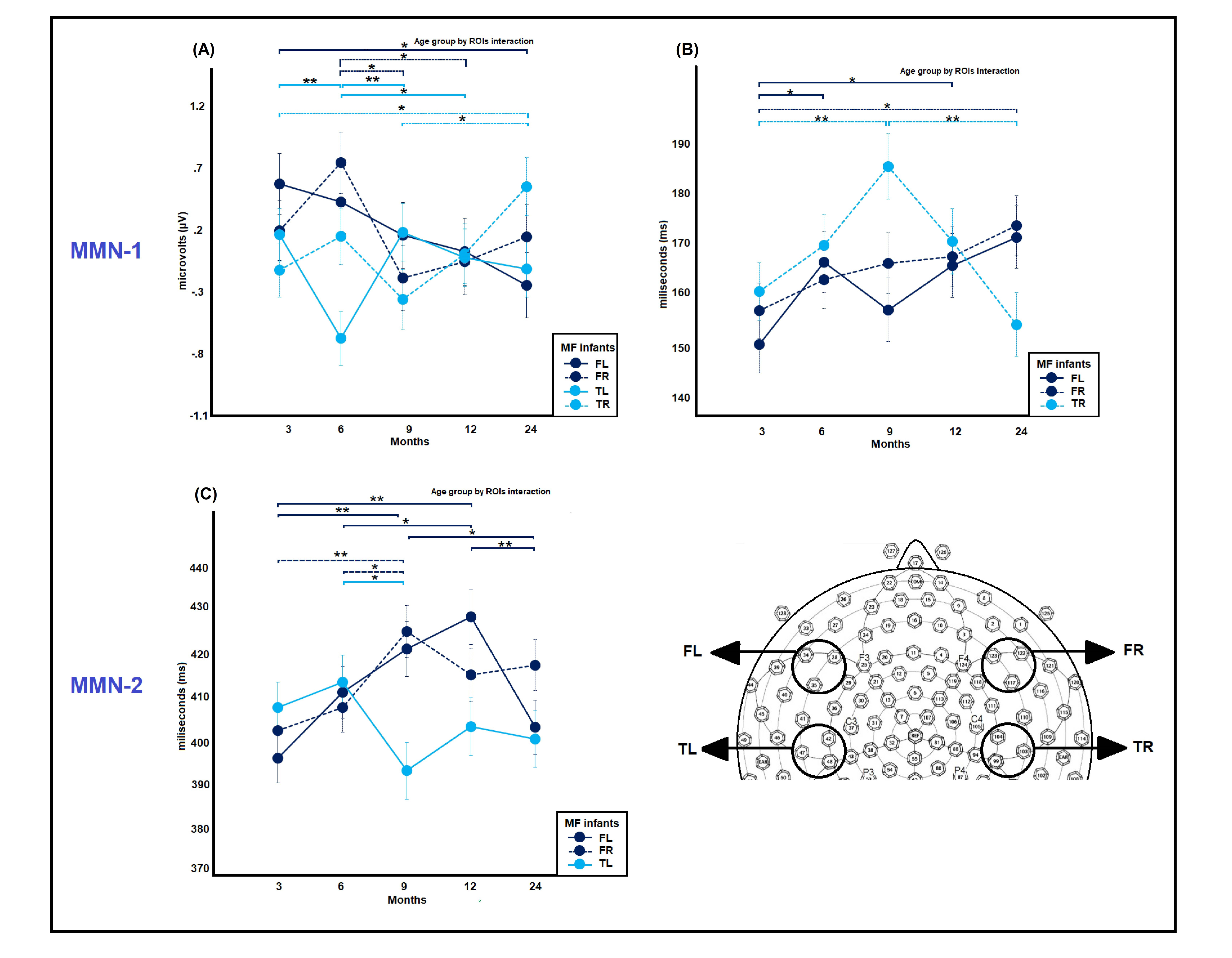

Supplement: Supplementary file 6 [file Image_2.JPEG]

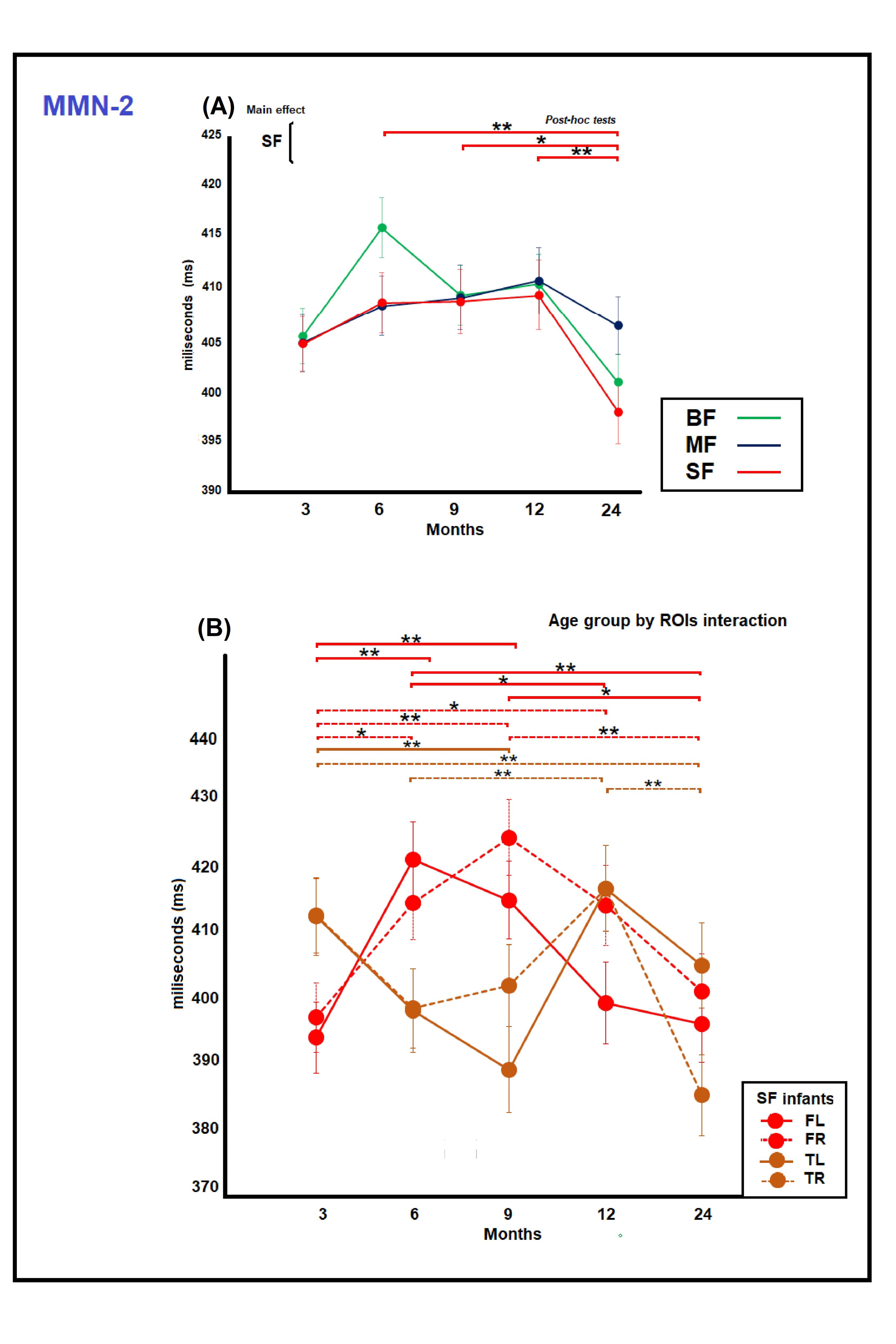

Supplement: Supplementary file 7 [file Image_3.JPEG]
